# Implementation of Laplace Transformed MP2 for Periodic Systems With Numerical Atomic Orbitals

**DOI:** 10.3389/fchem.2020.589992

**Published:** 2020-11-10

**Authors:** Honghui Shang, Jinlong Yang

**Affiliations:** ^1^State Key Laboratory of Computer Architecture, Institute of Computing Technology, Chinese Academy of Sciences, Beijing, China; ^2^Hefei National Laboratory for Physical Sciences at Microscale, Department of Chemical Physics, Synergetic Innovation Center of Quantum Information and Quantum Physics, University of Science and Technology of China, Hefei, China

**Keywords:** MP2, NAO, real-space, Hartree–Fock, periodic system

## Abstract

We present an implementation of the canonical and Laplace-transformed formulation of the second-order Møller–Plesset perturbation theory under periodic boundary conditions using numerical atomic orbitals. To validate our approach, we show that our results of the Laplace-transformed MP2 correlation correction for the total energy and the band gap are in excellent agreement with the results of the canonical MP2 formulation. We have calculated the binding energy curve for the stacked trans-polyacetylene at the Hartree–Fock + MP2 level as a preliminary application.

## 1. Introduction

The second-order Møller–Plesset perturbation theory (MP2) is a post-Hartree–Fock approach to take the electron correlation effect into account. Although it is very simple in form, it can capture around 90% of the correlation energy (Bartlett and Stanton, 2007); so the MP2 method is still of high interest in the quantum chemistry (Schütz et al., 1999; Kobayashi and Nakai, 2006; Bartlett and Stanton, 2007) and solid-state physics communities (Suhai, 1983, 1992; Sun and Bartlett, 1996; Pisani et al., 2008; Marsman et al., 2009; Schäfer et al., 2018).

However, the *O*(*N*^5^) calculation scaling of the original (canonical) MP2 method has limited the application of the MP2 method in large systems. A series of algorithms have been proposed to speed up the calculations, such as local MP2 method (Saebø and Pulay, 1993; Pisani et al., 2005, 2008; Maschio, 2011), Lapace-transformed MP2 method (Häser and Almlöf, 1992; Häser, 1993; Ayala and Scuseria, 1999; Ayala et al., 2001; Schäfer et al., 2018), or resolution of the identity (RI) MP2 method (Katouda and Nagase, 2010; Ren et al., 2012). The local MP2 method proposed by Pulay (1983) and Saebø and Pulay (1993) has been efficiently implemented (Schütz et al., 1999) in the MOLPRO code for molecules, then the periodic version of the local MP2 method has been implemented (Pisani et al., 2005, 2008; Maschio, 2011) in the CRYSCOR code and in the CP2K code (Usvyat et al., 2018) for extended systems. Since the spatially localized orbitals or Wannier functions are adopted, the computational scaling of the local MP2 method is *O*(*N*). The Laplace-transformed MP2 method is originally proposed by Häser and Almlöf (1992) and Häser (1993), and have been implemented for both the molecule (Ayala and Scuseria, 1999) and extended systems (Ayala et al., 2001) in the GAUSSIAN suite of programs. The localized atomic orbitals have been employed and the computational scaling is also O(N). The Laplace-transformed MP2 method has been combined with the resolution of identity (RI) technique to further improve the computational efficiency (Izmaylov and Scuseria, 2008). Further rigorous integral screening scheme (Lambrecht et al., 2005) has been introduced on top of the Laplace-transformed MP2 to perform the calculations for a system comprising 1,000 atoms (Doser et al., 2008). Recently, the Laplace-transformed MP2 method has also been implemented (Schäfer et al., 2018) in VASP using stochastic orbitals.

So far, most of the implementations of the MP2 are adopting the Gaussian-type orbital (GTO) as the basis set. However, in the calculation of the periodic system, too diffused GTO with a long tail will increase the number of cells in the auxiliary supercell, and therefore the computational cost will increase. Compared with GTO, the numerical atomic orbital (NAO) is strictly localized, which could naturally leads to lower order scaling of computational time vs. system size. Here in this work, we have implemented the canonical MP2 and Laplace-transformed MP2 for the extended systems using NAO, and the results obtained by these two approaches are consistent. Furthermore, we have investigated the MP2 correlation correction to the band structure with both the canonical and Laplace-transformed formulation; our implementation has been validated by comparing the MP2 correlation correction of the total energy and the band gap to the literature values.

The remainder of this paper is organized as follows. The fundamental theoretical framework and the implementation details for the canonical and Laplace transformed MP2 are presented in section 2. The benchmark calculations are presented in section 3. In section 4, we summarize our main achievement and highlight the possible future research direction related to this work.

## 2. Method

### 2.1. Numerical Atomic Orbitals

The numerical atomic orbital is defined by a product of a numerical radial function and a spherical harmonic

(1) $$\begin{array}{r} \chi_{Ilmn}\left(\text{r}\right)=\varphi_{Iln}\left(r\right){\text{Y}}_{lm}\left(\hat{r}\right). \end{array}$$

By solving the one-dimension radial Schrödinger equation

(2) $$\begin{array}{r} (-\frac{1}{2}\frac{1}{r}\frac{{\text{d}}^{2}}{\text{d}r^{2}}r+\frac{l(l+1)}{2r^{2}}+V(r)+V_{cut})\varphi_{Iln}(r)=\epsilon_{l}\varphi_{Iln}(r)\;, \end{array}$$

we can get the radial part of the numerical atomic orbital φ_*Iln*_(*r*), where *V*(*r*) denotes the electrostatic potential for orbital φ_*Iln*_(*r*), and *V*_*cut*_ ensures a smooth decay of each radial function, which is strictly zero outside a confining radius *r*_*cut*_.

In order to perform the Hartree–Fock and MP2 calculation, the electron repulsion integrals (ERIs) are needed:

(3) $$\begin{array}{r} (\chi_{\mu }\chi_{\nu }|\chi_{\lambda }\chi_{\sigma })=\int \int \frac{\chi_{\mu }(r)\chi_{\nu }(r)\chi_{\lambda }(r^{\prime })\chi_{\sigma }(r^{\prime })}{\left|r-r^{\prime }\right|}drdr^{\prime } \end{array}$$

we use NAO2GTO scheme described to calculate them as shown in the following section.

### 2.2. The NAO2GTO Scheme to Calculate ERIs

In the NAO2GTO scheme, we fit the NAO with GTOs, then we calculate the ERIs analytically; in this way, the strict cutoff of the atomic orbitals is satisfied with NAO and the construction of Hartree-Fock exchange (HFX) matrix can scale linearly with the system sizes (Shang et al., 2011). Since the angular part of the NAOs is spherical harmonic, while the GTOs are Cartesian harmonic function, a transformation between the Cartesian and spherical harmonic functions is performed within the NAO2GTO scheme.

### 2.3. Canonical MP2 Formulation

In extended systems, the normalized crystal orbital ψ_*i*_(**k, r**) is a linear combination of Bloch functions ϕ_μ_(**k, r**):

(4) $$\begin{array}{r} \psi_{i}\left(\text{k},\text{r}\right)=\underset{\mu }{\sum }C_{\mu ,i}\left(\text{k}\right)\phi_{\mu }\left(\text{k},\text{r}\right) \end{array}$$

(5) $$\begin{array}{r} \phi_{\mu }(k,r)=\frac{1}{\sqrt{N}}\sum_{R}\chi_{\mu }^{R}(r)e^{ik\cdot (R+r_{\mu })} \end{array}$$

in which *N* is the number of cells in extended systems, μ is the index of the atomic orbitals, *i* refers to the crystal orbital index, **R** denotes the cells in the extended systems (auxiliary supercell), $\chi_{\mu }^{\text{R}}\left(\text{r}\right)=\chi_{\mu }\left(\text{r}-\text{R}-{\text{r}}_{\mu }\right)$ refers to the atomic orbital whose center is displaced from the cell **R** by **r**_μ_, and *C*_μ, *i*_(**k**) are the coefficients of the crystal orbitals.

The MP2 correlation correction for the total energy of the unit cell is

(6) $$\begin{array}{ll} E_{\text{mp2}} & =-\frac{1}{N}\sum_{i}\sum_{j}\sum_{a}\sum_{b}\frac{1}{V_{k}^{4}}\int dk_{i}\int dk_{j}\int dk_{a}\\ \; & \int dk_{b}\frac{(IA|JB){\left[2(IA|JB)-(IB|JA)\right]}^{*}}{\epsilon_{a}(k_{a})+\epsilon_{b}(k_{b})-\epsilon_{i}(k_{i})-\epsilon_{j}(k_{j})} \end{array}$$

in which we use labeling i,j for occupied orbitals and a,b for unoccupied orbitals. *I/J* refer to the composite index (i,**k**_*i*_)/(j,**k**_*j*_), *V*_*k*_ is the volume of the Brillouin zone, and ϵ_*i*_(**k_i_**) is the Hartree–Fock eigenvalue for the eigenstate ψ_*i*_(**k_i_**). It should be noted that by using the identity ($\underset{\text{R}}{\sum }\exp i\text{k}\cdot \text{R}=N\delta_{\text{k},0}$) derived with the Born–von Karman periodic boundary condition, we can remove one dimension integration over **k**_*j*_ since **k**_*j*_ = **T**(−**k**_*i*_ + **k**_*a*_ + **k**_*b*_), where **T** is the translation operator. The formalism of summation over 3-fold **k** points and over 4-fold **k** points (Equation 6) give the same results.

Similarly, the MP2 correlation correction ($\epsilon_{g}{\left({\text{k}}_{g}\right)}^{\left(2\right)}$) to the Hartree–Fock eigenstate ψ_*g*_(**k**_*g*_) can be written as

(7) $$\begin{array}{r} \epsilon_{g}{\left({\text{k}}_{g}\right)}^{\left(2\right)}=\epsilon_{g}{\left({\text{k}}_{g}\right)}^{\text{MP2}}-\epsilon_{g}{\left({\text{k}}_{g}\right)}^{\text{HF}}=U\left(g\right)+V\left(g\right) \end{array}$$

(8) $$\begin{array}{r} U(g)=-\sum_{i,a,b}\frac{1}{V_{k}^{3}}\int dk_{i}\int dk_{a}\int dk_{b}\frac{(IA|GB){\left[2(IA|GB)-(IB|GA)\right]}^{*}}{\epsilon_{a}(k_{a})+\epsilon_{b}(k_{b})-\epsilon_{i}(k_{i})-\epsilon_{g}(k_{g})} \end{array}$$

(9) $$\begin{array}{r} V(g)=\sum_{i,j,a}\frac{1}{V_{k}^{3}}\int dk_{i}\int dk_{j}\int dk_{a}\frac{(IA|JG){\left[2(IA|JG)-(IG|JA)\right]}^{*}}{\epsilon_{a}(k_{a})+\epsilon_{g}(k_{g})-\epsilon_{i}(k_{i})-\epsilon_{j}(k_{j})} \end{array}$$

When ψ_*g*_(**k**_*g*_) is the occupied orbital at the valance band maximum (VBM), we can see *U*(*g*) < 0, *V*(*g*) > 0 and |*U*(*g*)| < |*V*(*g*)|, then the MP2 renormalization of the VBM is positive and will move the VBM orbital upward. When ψ_*g*_(**k**_*g*_) is the unoccupied orbital at the conduction band minimum (CBM), we have |*U*(*g*)| > |*V*(*g*)|, so the MP2 renormaliztion of the CBM is negative, and will move the CBM downward. In total, the MP2 renormalization of the band gap is negative, and the MP2 band gap is smaller than the Hartree-Fock band gap.

### 2.4. Laplace-Transformed MP2 Formulation

The Laplace transform is defined as:

(10) $$\begin{array}{r} \frac{1}{x}=\int_{0}^{\infty }e^{-xt}\text{d}t,x>0 \end{array}$$

which can be used to remove the denominator in the canonical MP2 formulation:

(11) $$\begin{array}{r} \frac{1}{\epsilon_{a}\left(k_{a}\right)+\epsilon_{b}\left(k_{b}\right)-\epsilon_{i}\left(k_{i}\right)-\epsilon_{j}\left(k_{j}\right)}=\int e^{\left[\epsilon_{i}\left(k_{i}\right)+\epsilon_{j}\left(k_{j}\right)-\epsilon_{a}\left(k_{a}\right)-\epsilon_{b}\left(k_{b}\right)\right]t}\text{d}t \end{array}$$

The integration in Equation (10) can either be done by using a least square fitting method (Häser and Almlöf, 1992; Häser, 1993) or by using a Jacobian transform (Ayala and Scuseria, 1999; Kobayashi and Nakai, 2006) of the Laplace integration variable in order to transform the integration range [0, ∞) into the finite range [0, 1]. Here, we use the transform as follows:

(12) $$\begin{array}{r} \int_{0}^{\infty }e^{-xt}\text{d}t=\int_{0}^{1}e^{-xt}\frac{\text{d}t}{\text{d}r}\text{d}r=\int_{0}^{1}f\left(r\right)\text{d}r \end{array}$$

in which the Jacobian transform is

(13) $$\begin{array}{r} t=\frac{r^{3}-0.9r^{4}}{{\left(1-r\right)}^{2}}+r^{2}\tan\left(\frac{\pi r}{2}\right) \end{array}$$

Then the final integration ($\int_{0}^{1}f\left(r\right)\text{d}r$) in Equation (12) in evaluated with Romberg quadrature method, which uses refinements of the extended trapezoidal rule to reduce error in definite integrals.

In this way, the *E*_mp2_ correlation correction energy can be written as a new integration form:

(14) $$\begin{array}{l} E_{\text{mp2}}=-\int dt\sum_{\mu 0,\nu R_{\nu },\lambda R_{\lambda },\sigma R_{\sigma }}T_{\mu ,\lambda ,\nu ,\sigma }^{0R_{\lambda }R_{\nu }R_{\sigma }}(t)[2(\chi_{\mu }^{0}\chi_{\lambda }^{R_{\lambda }}|\chi_{\nu }^{R_{\nu }}\chi_{\sigma }^{R_{\sigma }})\\ \;-(\chi_{\mu }^{0}\chi_{\sigma }^{R_{\sigma }}|\chi_{\nu }^{R_{\nu }}\chi_{\lambda }^{R_{\lambda }})] \end{array}$$

where μ,ν,σ,λ and the following λ,δ,τ,κ refer to the indexes of the atomic orbitals. $\left(\chi_{\mu }^{0}\chi_{\sigma }^{{\text{R}}_{\sigma }}|\chi_{\nu }^{{\text{R}}_{\nu }}\chi_{\lambda }^{{\text{R}}_{\lambda }}\right)$ is the electron repulsion integrals defined as

(15) $$\begin{array}{r} (\chi_{\mu }^{0}\chi_{\sigma }^{R_{\sigma }}|\chi_{\nu }^{R_{\nu }}\chi_{\lambda }^{R_{\lambda }})=\int \int \frac{\chi_{\mu }^{0}(r)\chi_{\sigma }^{R_{\sigma }}(r)\chi_{\nu }^{R_{\nu }}(r^{\prime })\chi_{\lambda }^{R_{\lambda }}(r^{\prime })}{\left|r-r^{\prime }\right|}drdr^{\prime }\;. \end{array}$$

and

(16) $$\begin{array}{rc} T_{\mu ,\lambda ,\nu ,\sigma }^{{\text{R}}_{\mu }{\text{R}}_{\lambda }{\text{R}}_{\nu }{\text{R}}_{\sigma }}\left(t\right)=\\ \underset{\gamma {\text{R}}_{\gamma },\delta {\text{R}}_{\delta },\tau {\text{R}}_{\tau },\kappa {\text{R}}_{\kappa }}{\sum } & X_{\mu \gamma }^{{\text{R}}_{\mu }{\text{R}}_{\gamma }}X_{\nu \delta }^{{\text{R}}_{\nu }{\text{R}}_{\delta }}Y_{\lambda \kappa }^{{\text{R}}_{\lambda }{\text{R}}_{\kappa }}Y_{\sigma \tau }^{{\text{R}}_{\sigma }{\text{R}}_{\tau }}\left(\chi_{\gamma }^{{\text{R}}_{\gamma }}\chi_{\kappa }^{{\text{R}}_{\kappa }}|\chi_{\delta }^{{\text{R}}_{\delta }}\chi_{\tau }^{{\text{R}}_{\tau }}\right) \end{array}$$

The 4-fold k points are treated independently within $X_{\gamma \mu }^{{\text{R}}_{\gamma }{\text{R}}_{\mu }}$ and $Y_{\lambda \kappa }^{{\text{R}}_{\lambda }{\text{R}}_{\kappa }}$:

(17) $$\begin{array}{r} X_{\gamma \mu }^{R_{\gamma }R_{\mu }}=\sum_{i}^{occ}\frac{1}{V_{k}}\int dk_{i}C_{\gamma i}^{*}(k_{i})C_{\mu i}(k_{i})e^{(\epsilon_{i}-\epsilon_{f})t}e^{ik_{i}(R_{\mu }-R_{\gamma })} \end{array}$$

(18) $$\begin{array}{r} Y_{\lambda \kappa }^{R_{\lambda }R_{\kappa }}=\sum_{a}^{unocc}\frac{1}{V_{k}}\int dk_{a}C_{\lambda a}^{*}(k_{a})C_{\kappa a}(k_{a})e^{-(\epsilon_{a}-\epsilon_{f})t}e^{ik_{a}(R_{\kappa }-R_{\lambda })} \end{array}$$

In this way, the 4-fold integration over **k**-points in Equation (6) can be reduced to 1-dimensional **k**-points integral as shown in Equations (17) and (18). Furthermore, the locality of the atomic basis function can be adopted for the calculation of $T_{\mu ,\lambda ,\nu ,\sigma }^{{\text{R}}_{\mu }{\text{R}}_{\lambda }{\text{R}}_{\nu }{\text{R}}_{\sigma }}$ and electron repulsion integrals, and the total computational scaling could be *O*(*N* · *N*_*k*_) if the distant screening between these ERIs are applied. Here in this work, such distance screening has not been used, so our implementation results in a $O\left(N^{2}\cdot N_{k}\right)$ scaling. It is worth noting that in order to keep the exponential value ($e^{\left(\epsilon_{i}\right)t}$) in Equation (17)/Equation (18) to be smaller than unity, we have inserted the Fermi energy level into the exponential factor ($e^{\left(\epsilon_{i}-\epsilon_{f}\right)t}$) in order to make the calculation to be more stable.

Based on Equations (7) and (10), we have the Laplace-transformed MP2 correlation correction ($\epsilon_{g}{\left({\text{k}}_{g}\right)}^{\left(2\right)}$) for the eigenstate:

(19) $$\begin{array}{ll} \epsilon_{g}{\left(k_{g}\right)}^{\left(2\right)} & =\int dt\sum_{\mu 0,\nu R_{\nu },\lambda R_{\lambda },\sigma R_{\sigma }}G_{\mu ,\lambda ,\nu ,\sigma }^{0R_{\nu }R_{\lambda }R_{\sigma }}(t)[2(\chi_{\mu }^{0}\chi_{\lambda }^{R_{\lambda }}|\chi_{\nu }^{R_{\nu }}\chi_{\sigma }^{R_{\sigma }})\\ \; & \;-(\chi_{\mu }^{0}\chi_{\sigma }^{R_{\sigma }}|\chi_{\nu }^{R_{\nu }}\chi_{\lambda }^{R_{\lambda }})] \end{array}$$

(20) $$\begin{array}{l} G_{\mu ,\lambda ,\nu ,\sigma }^{{\text{R}}_{\mu }{\text{R}}_{\lambda }{\text{R}}_{\nu }{\text{R}}_{\sigma }}\left(t\right)=\underset{\gamma {\text{R}}_{\gamma },\delta {\text{R}}_{\delta },\tau {\text{R}}_{\tau },\kappa {\text{R}}_{\kappa }}{\sum }X_{\mu \gamma }^{{\text{R}}_{\mu }{\text{R}}_{\gamma }}Y_{\lambda \kappa }^{{\text{R}}_{\lambda }{\text{R}}_{\kappa }}\left(\chi_{\gamma }^{{\text{R}}_{\gamma }}\chi_{\kappa }^{{\text{R}}_{\kappa }}|\chi_{\delta }^{{\text{R}}_{\delta }}\chi_{\tau }^{{\text{R}}_{\tau }}\right)\\ \;\times \left(-W_{\nu \delta }^{{\text{R}}_{\nu }{\text{R}}_{\delta }}Y_{\sigma \tau }^{{\text{R}}_{\sigma }{\text{R}}_{\tau }}+X_{\nu \delta }^{{\text{R}}_{\nu }{\text{R}}_{\delta }}Z_{\sigma \tau }^{{\text{R}}_{\sigma }{\text{R}}_{\tau }}\right) \end{array}$$

(21) $$\begin{array}{r} W_{\gamma \mu }^{{\text{R}}_{\gamma }{\text{R}}_{\mu }}=C_{\gamma g}^{*}\left({\text{k}}_{g}\right)C_{\mu g}\left({\text{k}}_{g}\right)e^{\left(\epsilon_{g}\right)t}e^{i{\text{k}}_{g}\left({\text{R}}_{\mu }-{\text{R}}_{\gamma }\right)} \end{array}$$

(22) $$\begin{array}{r} Z_{\lambda \kappa }^{{\text{R}}_{\lambda }{\text{R}}_{\kappa }}=C_{\lambda g}^{*}\left({\text{k}}_{g}\right)C_{\kappa g}\left({\text{k}}_{g}\right)e^{-\left(\epsilon_{g}\right)t}e^{i{\text{k}}_{g}\left({\text{R}}_{\kappa }-{\text{R}}_{\lambda }\right)} \end{array}$$

Similarly, in order to keep the exponential value to be smaller than unity and avoid computational divergence, we inserted the VBM/CBM value into the exponential factor when calculated the MP2 reformulation of the VBM/CBM.

The canonical and Laplace-transformed MP2 methods described above have been implemented in the Order-N performance HONPAS code (Qin et al., 2014).

## 3. Results

In order to validate our implementation, we perform benchmark calculations for 1-dimensional systems. We use norm-conserving pseudopotentials generated with the Troullier–Martins scheme to represent the interaction between core ion and valence electrons. The single-zeta (SZ), double-zeta (DZ), and double-zeta polarized (DZP) basis sets are generated using SIESTA. Then the NAOs are fitted with GTOs to perform the Hartree–Fock calculation as discussed in Shang et al. (2011).

First, we use a 1-dimensional hydrogen chain as an example to make the comparison between the results of canonical MP2 and those of Laplace-transformed MP2. The lattice parameter for the 1-dimensional H chain is set to 2.6 Å, and the H-H bond length is set to 1.346 Å. The SZ basis set is adopted, so that there are only two atomic orbitals in the unit cell. The Brillouin zone is sampled by 1 × 1 × 6 k-points. The unit cell is a 20 × 20 × 2.6 Åbox, and the real-space integration mesh is set to be 100 Ry. In the Laplace-transformed MP2 method, the Romberg method is adopted to perform the final integration. The accuracy of integration results depends on the order of Romberg integration (n), since the results of the Romberg integration are obtained in a recursive manner, $R\left(n,j\right)=R\left(n,j-1\right)+\frac{R\left(n,j-1\right)-R\left(n-1,j-1\right)}{4^{j-1}-1}$. As shown in Table 1, when the order of Romberg integration increases from 3 to 8, the MP2 correlation correction for the unit cell energy (*E*_mp2_) as well as the MP2 correlation correction for the band structure ($\epsilon_{\text{VBM}}^{\left(2\right)}$, $\epsilon_{\text{CBM}}^{\left(2\right)}$, $\epsilon_{\text{gap}}^{\left(2\right)}$) are converged to the results of canonical MP2. When using the media precision parameter (*n* = 5) in Laplace- transformed MP2, we get a absolute/relative error of 4 × 10^−6^ eV/0.0007% for the correlation of the unit cell energy (*E*_mp2_), and we get a absolute/relative error of 3 × 10^−5^ eV/0.02% for the MP2 correlation correction for the band gap ($\epsilon_{\text{gap}}^{\left(2\right)}$). Overall, we find an excellent agreement between the Laplace-transformed MP2 and the canonical MP2 benchmark results.

**Table 1:** The comparison between the results of canonical MP2 and those of Laplace-transformed MP2.

**H2-line**		**Laplace**		**Canonical**
***n***	**3**	**5**	**8**
*E*_mp2_ (eV)	−0.561442	−0.561190	−0.561194	−0.561194
$\epsilon_{\text{VBM}}^{\left(2\right)}$ (eV)	0.16736223	0.150361892	0.15033577	0.15033577
$\epsilon_{\text{CBM}}^{\left(2\right)}$ (eV)	−0.03044718	−0.01888047	−0.01887073	−0.01887073
$\epsilon_{\text{gap}}^{\left(2\right)}$ (eV)	−0.19780941	−0.16924236	−0.16920650	−0.16920650

*Here, the MP2 correlation correction for the unit cell energy (E_mp2_) and the MP2 correlation correction for the band structure ($\epsilon_{\text{VBM}}^{\left(2\right)}$, $\epsilon_{\text{CBM}}^{\left(2\right)}$, $\epsilon_{\text{gap}}^{\left(2\right)}$) have been examined with the above two approaches. Here, we use a 1-dimensional hydrogen chain as an example*.

We also examine the relative error between the results of Laplace-transformed MP2 and those of canonical MP2 with respect to the basis set size (SZ, DZ, DZP), as shown in Table 2 with ethylene molecule as an example. Again, we find an excellent agreement between the Laplace-transformed MP2 and the canonical MP2 benchmark results.

**Table 2:** The relative error between the results of canonical MP2 and those of Laplace-transformed MP2 with different basis set.

**Ethylene (C_**2**_H_**4**_)**	**SZ (%)**	**DZ (%)**	**DZP (%)**
Relative error	0.0007	0.003	0.001

*Here, we use ethylene molecule as an example. The order of Romberg integration (n) in the Laplace transformed MP2 is set to n = 5*.

Second, we perform the Laplace-transformed MP2 calculation for the 1D polymer trans-polyacetylene as shown in Table 3. The order of Romberg integration is set to be *n* = 5. The SZ basis set is adopted in our calculation. The Brillouin zone is sampled by 1 × 1 × 30 k-points. The real-space integration mesh is set to be 200 Ry. We compare our calculated MP2 correlation correction for the total energy per unit cell with the one obtained in Sun and Bartlett (1996). The G3 geometry parameters as listed in Sun and Bartlett (1996) are adopted to keep the geometry to be the same for comparison. We get an absolute/relative error of 0.26 eV/8% for the correlation correction of the unit cell energy (*E*_mp2_). The difference comes from the usage of the different basis set, since in Sun and Bartlett (1996), the STO-3G basis set is used, whose shape is different from the SZ basis that we are using. For a similar reason, when using the same G6 geometry parameter of trans-polyacetylene (Sun and Bartlett, 1996), we get an absolute/relative error of 0.09 eV/7% for the correlation correction of the band gap ($\epsilon_{\text{gap}}^{\left(2\right)}$) when compared with Sun's result.

**Table 3:** The comparison between our results and those from the literature (Sun and Bartlett, 1996).

**Trans-polyacetylene**	**Sun and Bartlett (1996) (eV)**	**Our results (eV)**
*E*_mp2_	−3.22	−2.96
$\epsilon_{\text{gap}}^{\left(2\right)}$	−1.18	−1.09

*Here, the MP2 correlation correction for the unit cell energy (E_mp2_) and the MP2 correlation correction for the band gap ($\epsilon_{\text{gap}}^{\left(2\right)}$) have been examined*.

**Figure 1:**
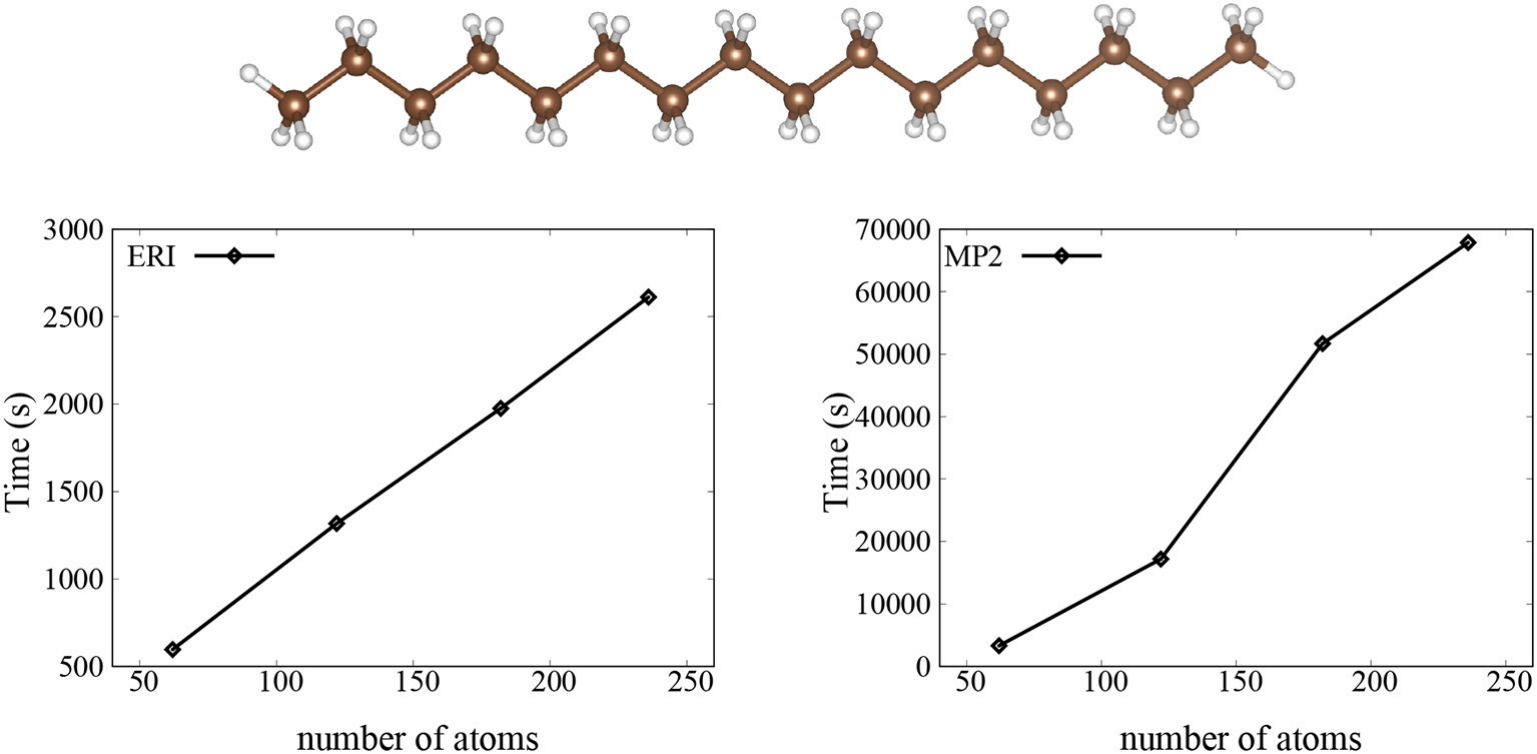
The CPU time for the ERI and MP2 calculation using SZ basis set. Here, the trans-polyacetylene molecules are used as the test systems.

We then investigate the performance and scaling of our implementation, and we show timings for the trans-polyacetylene molecules with variable number of atoms in Figure 1. We find a linear scaling for the calculation of ERIs and an O(N^2^) scaling for the calculation of the Laplace-transformed MP2. This is not too surprising, since we can see from Equation (14) that there are two loops over the ERIs for the calculation of Laplace-transformed MP2, so we get the O(N^2^) scaling.

**Figure 2:**
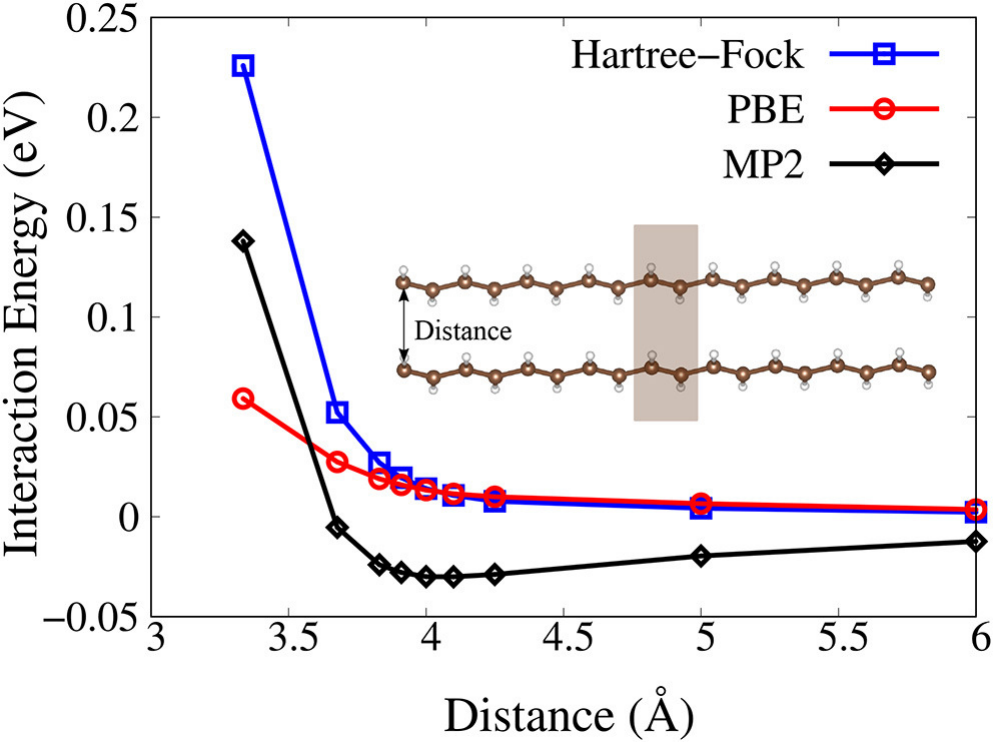
Interaction energy as functions of the distance between two trans-polyacetylene chains as predicted by the Hartree-Fock (blue), PBE (red), and MP2 (black) method. The unit cell is marked with shaded box.

Finally, we show the calculated binding-energy curves as functions of the distance between two trans-polyacetylene chains with the PBC-MP2 method. Although the MP2 theory gives overestimation of the dispersion interaction energy (Tkatchenko et al., 2009), it is still a superior starting point for the dispersion correction compared to Hartree–Fock and semi-local density functional theory (DFT). As shown in Figure 2, the MP2 method results in a binding states. On the contrary, Hartree–Fock and PBE functional fail to identify any binding between the two chains. We can see in Figure 2 that the energy profile calculated with Hartree–Fock and PBE functional shows a repulsive behavior as the two chains are brought closer together.

## 4. Conclusions

We have implemented the canonical and Laplace-transformed algorithms to calculate the MP2 correlation correction for the total energy and the band gap of periodic systems in HONPAS code with numerical atomic orbitals. The results obtained by the canonical MP2 and Laplace-transformed MP2 are consistent with each other. We have also validated the implementation by comparing the results with the literature data. We have studied the binding-energy curves for the two stacked trans-polyacetylene chains, which shows the MP2 method can well describe the correlation energy and the long-range van der Waals interactions. Future work will address the application of the Laplace-transformed MP2 method to 3-dimensional periodic systems in the HONPAS code.

## Data Availability Statement

All datasets generated for this study are included in the article/supplementary material.

## Author Contributions

JY designed and directed the project. HS developed the theoretical formalism, carried out the implementation, and performed the numerical simulations. Both JY and HS contributed to the final version of the manuscript. All authors contributed to the article and approved the submitted version.

## Conflict of Interest

The authors declare that the research was conducted in the absence of any commercial or financial relationships that could be construed as a potential conflict of interest.
